# Genetic Liability to Insomnia and Lung Cancer Risk: A Mendelian Randomization Analysis

**DOI:** 10.3389/fgene.2021.756908

**Published:** 2021-12-01

**Authors:** Jiayi Shen, Huaqiang Zhou, Jiaqing Liu, Yaxiong Zhang, Ting Zhou, Gang Chen, Wenfeng Fang, Yunpeng Yang, Yan Huang, Li Zhang

**Affiliations:** ^1^ Department of Medical Oncology, Sun Yat-sen University Cancer Center, Guangzhou, China; ^2^ State Key Laboratory of Oncology in South China, Guangzhou, China; ^3^ Collaborative Innovation Center for Cancer Medicine, Guangzhou, China; ^4^ Zhongshan School of Medicine, Sun Yat-sen University, Guangzhou, China

**Keywords:** insomnia, lung cancer, mendelian randomization analysis, causality, prevention

## Abstract

Lung cancer is the second most frequently diagnosed cancer and the leading cause of cancer death worldwide, making its prevention an urgent issue. Meanwhile, the estimated prevalence of insomnia was as high as 30% globally. Research on the causal effect of insomnia on lung cancer incidence is still lacking. In this study, we aimed to assess the causality between the genetic liability to insomnia and lung cancer. We performed a two-sample Mendelian randomization analysis (inverse variance weighted) to determine the causality between the genetic liability to insomnia and lung cancer. Subgroup analysis was conducted, which included lung adenocarcinoma and lung squamous cell carcinoma. In the sensitivity analysis, we conducted heterogeneity test, MR Egger, single SNP analysis, leave-one-out analysis, and MR PRESSO. There were causalities between the genetic susceptibility to insomnia and increased incidence of lung cancer [odds ratio (95% confidence interval), 1.35 (1.14–1.59); *P*, < 0.001], lung adenocarcinoma [odds ratio (95% confidence interval), 1.35 (1.07–1.70); *P*, 0.01], and lung squamous cell carcinoma [odds ratio (95% confidence interval), 1.35 (1.06–1.72), *P*, 0.02]. No violation of Mendelian randomization assumptions was observed in the sensitivity analysis. There was a causal relationship between the genetic susceptibility to insomnia and the lung cancer, which was also observed in lung adenocarcinoma and lung squamous cell carcinoma. The underlying mechanism remains unknown. Effective intervention and management for insomnia were recommended to improve the sleep quality and to prevent lung cancer. Moreover, regular screening for lung cancer may be beneficial for patients with insomnia.

## Introduction

Lung cancer is the second most frequently diagnosed cancer for both male and female in the world, of which the estimated number of new cases was 228,150 in 2019 (Siegel et al., 2019). It is also the leading cause of cancer death worldwide, with the estimated number of new deaths as 142,670 in 2019. Regarding its high incidence and mortality, lung cancer has long been a heavy burden in public health, making lung cancer prevention an urgent issue. For this reason, it is meaningful to investigate whether there are causalities between potential risk factors and lung cancer, to provide guidance in lung cancer prevention.

Insomnia has become a common sleep disorder worldwide, with the estimated prevalence as 30% (Roth, 2007). Previous studies mainly revealed the association between poor sleep habits like prolonged or shortened sleep duration and cancer incidence (Kakizaki et al., 2008; Chen et al., 2019). The incidence of lung cancer increased when sleep duration was ≤6.5 h or ≥8 h(Luojus et al., 2014). However, insomnia disorder is not simply characterized by reduced sleep duration but more by difficulties falling asleep and sleep disturbance (Morin et al., 2015). Research focusing on the causal effect of insomnia on lung cancer incidence is still lacking. We think it necessary to analyze the causality between insomnia and lung cancer, considering the urgency of lung cancer prevention, high prevalence and the potential carcinogenicity of insomnia.

Mendelian randomization (MR) analysis is a novel epidemiological approach for the estimation of causality between exposure and outcome (Smith and Ebrahim, 2003). In MR analysis, single-nucleotide polymorphisms (SNPs), which have been identified to be robustly correlated with the exposure, are used as proxies of exposure. SNPs of exposure should be correlated with the risk of outcome to the extent predicted by their influence on exposure, if the causality between exposure and outcome exists (Smith et al., 2008). MR analysis can be a potential mimic of randomized controlled trial (RCT) by utilizing SNPs, especially when RCT is too costly or infeasible (Smith and Ebrahim, 2004). SNPs, instrument variables in MR analysis, are randomly allocated during gamete formation and fertilization in the population, which is similar to the randomization in RCT. In this way, biases from confounders and inverse causality can also be avoided in MR analysis, which are common in observational studies (Davey Smith and Hemani, 2014).

In this study, we aimed to assess the causality between genetic liability to insomnia and lung cancer, utilizing two-sample MR analysis. We present the following article in accordance with the STROBE reporting checklist.

## Material and Methods

### Summary Data From Genome-wide Association Study on Insomnia and Lung Cancer

In a meta-analysis by Jansen et al., 248 SNPs were identified to be robustly correlated with insomnia (Jansen et al., 2019). Data from United Kingdom Biobank (UKB) version 2 (*n* = 386,533) and 23andMe (*n* = 944,477) were pooled. Sample size in total was 1,331 010. (Table 1). Information about insomnia was collected utilizing a self-report sleep questionnaire. Prevalence of insomnia in the combined sample was 29.9%. The questionnaires used by UKB and 23andMe were with high accuracy (sensitivity/specificity of UKB = 98/96%; sensitivity/specificity of 23andMe = 84/80%). The 248 SNPs were genome-wide significant (P < 5 × 10^−8^). These 248 SNPs were in linkage equilibrium with each other at *r* ^2^ < 0.1, and they could explain 2.6% of the variance in insomnia. Conclusively, the 248 SNPs can serve as the genetic instrumental variables for insomnia with enough statistical power. These 248 SNPs were utilized as the proxies of insomnia in this MR analysis.

**TABLE 1:** Genome-wide Association Study Utilized in this MR Analysis.

Consortium	Phenotype	Participants	Web source
CTGlab of CNCR	Insomnia	133,1010	https://ctg.cncr.nl/software/summary_statistics
ILCCO	Lung cancer, LUAD, LUSQ	27,209	ilcco.iarc.fr

CTGlab, complex trait genetics lab; CNCR, center for neurogenomics and cognitive research; ILCCO, international lung cancer consortium; LUAD, lung adenocarcinoma; LUSQ, lung squamous cell carcinoma.

We used summary data from a Genome-wide Association Study (GWAS) by International Lung Cancer Consortium (ILCCO) on lung cancer (11,348 cases and 15,861 controls), lung adenocarcinoma (LUAD) (3,442 cases and 14,894 controls), and lung squamous cell carcinoma (LUSQ) (3,275 cases and 15,038 controls). (Table 1) (Wang et al., 2014). The effects of the SNPs of insomnia on lung cancer, LUAD and LUSQ, the effect size and standard error, were extracted from the GWAS by ILCCO in the form of summary data through MR-base (Hemani et al., 2018). SNPs Summary data of insomnia and the outcomes were harmonized, where effect of each SNP on insomnia and outcomes were estimated and 12 SNPs were removed for being palindromic with intermediate allele frequencies (rs11126082, rs12454003, rs12991815, rs1731951, rs2030672, rs2221119, rs4858708, rs6545798, rs7044885, rs8180817, rs9373590, rs9540729). (Supplementary Table S1) Effect allele and frequency of effect allele were also provided.

To assess the potential existence of weak instrumental bias in this MR study, we also calculated the statistical power and F statistic of this study, with four presumed and fixed range of odds ratio (OR) (Brion et al., 2013) (Table 2). Power and F statistic were dependent on the strength of association between the SNPs and insomnia and the sample size of the outcome GWAS studies. The larger the power and F statistics were, the smaller the possibility of weak instrumental bias would be (cut off value for judgement, power, 80%; F statistic, 10) Powers were larger than 80%, only when OR was set to be “0.75 or 1.33” and “0.67 or 1.50” for lung cancer and when ORs were set to be “0.67 or 1.50” for LUAD and LUSQ. F statistics were far greater than 10 for lung cancer, LUAD and LUSQ.

**TABLE 2:** Power and F statistic for Conventional Mendelian Randomization Analysis (two-sided *α* = 0.05).

Outcome	Sample size of GWAS on outcome	Proportion of cases	Power to identify OR of 0.91 or 1.10	Power to identify OR of 0.83 or 1.20	Power to identify OR of 0.75 or 1.33	Power to identify OR of 0.67 or 1.50	F Statistic
Lung cancer	27,209	0.4171	0.24	0.68	0.96	1.00	727.32
LUAD	18,336	0.1877	0.13	0.38	0.61	0.98	490.46
LUSQ	18,313	0.1788	0.13	0.37	0.59	0.97	489.85

GWAS, genome-wide association study; OR, odds ratio; LUAD, lung adenocarcinoma; LUSQ, lung squamous cell carcinoma.

No patients were involved in the study design. Recruitment or conduct and the need for ethical approval was waived.

### Two-Sample MR Analysis

Two-sample MR was utilized to investigate the causality between the genetic liability to insomnia and lung cancer incidence. Two-sample MR can improve statistical power, with the utilization of summary data of SNPs from large scale GWAS (Burgess et al., 2015; Lawlor, 2016). In two-sample MR, the effects of SNPs of exposure on exposure and outcome are derived from GWAS of exposure and GWAS of outcome respectively. Specifically, inverse variance weighted (IVW) was used (Hemani et al., 2018). Subgroup analyses of LUAD and LUSQ were conducted to assess whether there was a difference between the MR estimate of lung cancer and those of LUAD and LUSQ, considering the reported difference in the etiologies between LUAD and LUSQ (Herbst et al., 2018).

Genetic instrumental variable used in MR analysis must fulfill three assumptions: 1) the instrumental variable is associated with the exposure; 2) the instrumental variable is associated with the outcome through the studied exposure merely; and 3) the instrumental variable is independent of other factors which affect the outcome (Boef et al., 2015). In terms of sensitivity analysis, we produced a MR regression slopes chart to display the difference between the result of IVW and those of MR Egger and weighted median. Additionally, the heterogeneity test was also conducted by performing Cochran’s Q test on the IVW and the MR-Egger estimate. If there is heterogeneity (Cochrane’s Q *p*-value < 0.05) and a random effect model was employed to it. To assess whether the assumptions of MR were violated, MR-Egger analysis was performed to detect directional horizontal pleiotropy, and a funnel plot was also generated (Bowden et al., 2015; Burgess and Thompson, 2017). The existence of pleiotropy means the instrument variable can be associated with the observed outcome through other mechanisms than insomnia. Furthermore, the detection of directional horizontal pleiotropy suggests that the sum of pleiotropy does not equal to zero, which means the violation of the 2) assumption (exclusion restriction assumption). If the intercept is close to 0 and *P* is close to one in MR-Egger analysis, the MR study will be free of directional horizontal pleiotropy. Single SNP analysis and leave-one-out analysis was performed to assess whether the result was driven by a single SNP. MR PRESSO was also conducted for the estimation of horizontal pleiotropy, which included global test, outlier test, and distortion test (Verbanck et al., 2018). If a horizontal pleiotropy was detected by the global test, the outlier test would be performed, figuring out the outlying SNPs. Subsequently, an outlier-corrected causal estimate would be assessed and compared with the original MR estimate, which was the distortion test, providing a *p*-value for the comparison.

The statistical analysis was performed utilizing the package TwoSampleMR (version 0.4.25) in R (version 3.6.1). The study was conducted in accordance with the Declaration of Helsinki (as revised in 2013).

## Results

### Causality From Insomnia to Lung Cancer

The genetic susceptibility to insomnia was causally associated with increased lung cancer incidence based on the results of IVW method {OR [95% confidence interval (CI)], 1.35 (1.14–1.59); *p* < 0.001; Cochrane’s Q *p*-value = 0.00078}. (Table 3). MR regression slopes showed positive correlation between the effect of SNP on insomnia and that on lung cancer. (Supplementary Figure S1) The three slopes according to the three different MR analyses were close to each other. Single SNP analysis indicated that the MR estimate of single SNP varied from each other. (Supplementary Table S2; Supplementary Figure S2) However, results in the leave-one-out analysis were similar to each other, indicating that there was no driving SNP in this MR analysis (Supplementary Table S3; Supplementary Figure S3) According to the result of MR-Egger analysis, directional horizontal pleiotropy was not detected, which meant the SNPs of insomnia did not affect the incidence of lung cancer through other traits than insomnia. (Table 4). The funnel plot was symmetrical, with the indication of no directional horizontal pleiotropy (Supplementary Figure S4) Horizontal pleiotropy and outlying SNPs were identified in the MR PRESSO global test, while the distortion test did not show a statistically significant difference between the original MR estimate and the outlier-corrected MR estimate. (Table 5).

**TABLE 3:** Mendelian randomization estimates of the causality between insomnia and lung cancer.

Exposure	Outcome	Inverse variance weighted	
		Or (95%CI)	*p*-value
Insomnia	Lung Cancer	1.35 (1.14–1.59)	<0.001
Insomnia	LUAD	1.35 (1.07–1.70)	0.01
Insomnia	LUSQ	1.35 (1.06–1.72)	0.02

OR, odds ratio; CI, confidence interval; LUAD, lung adenocarcinoma; LUSQ, lung squamous cell carcinoma.

**TABLE 4:** Results of MR Egger for the estimation of directional horizontal pleiotropy.

Outcome	Intercept	Standard error	*p*-value
Lung cancer	−0.001	0.006	0.91
Lung adenocarcinoma	0.004	0.009	0.64
Lung squamous cell carcinoma	0.005	0.009	0.62

**TABLE 5:** Results of MR PRESSO for the estimation of horizontal pleiotropy.

Outcome	*p*-value of global test	Outlying SNP	Outlier-corrected causal estimate	*p*-value of outlier-corrected causal estimate	*p*-value of distortion test
Lung cancer	<0.001	rs73079014, rs76145129	0.269	<0.001	0.61
LUAD	0.03	No significant outliers	NA	NA	NA
LUSQ	0.01	rs76145129	0.284	0.02	0.83

LUAD, lung adenocarcinoma; LUSQ, lung squamous cell carcinoma.

### Causal Effect From Insomnia to LUAD and LUSQ

According to the result of IVW, insomnia was positively correlated with the incidence of LUAD [OR (95% CI), 1.35 (1.07–1.70); *P*, 0.01; Cochrane’s Q *p*-value = 0.056] and LUSQ [OR (95% CI), 1.35 (1.06–1.72); *P*, 0.02; Cochrane’s Q *p*-value = 0.0050]. (Table 3) The MR regression slopes of both LUAD and LUSQ showed positive associations between the SNP effect on insomnia and the SNP effect on outcomes (Supplementary Figures S5; Supplementary Figure S6) However, the three regression curves did not overlap with each other, as were displayed in the two Supplementary Figures. Like the results of lung cancer, single SNP analysis of LUAD and LUSQ indicated varied MR effect size of single SNP (Supplementary Table S2; Supplementary Figure S7; Supplementary Figure S8), while leave-one-out analysis did not show signs of driving SNPs (Supplementary Table S3; Supplementary Figure S9; Supplementary Figure S10) MR Egger detected no directional horizontal pleiotropy for LUAD and LUSQ. (Table 4). Funnel plots of LUAD and LUSQ were symmetric, in support of the intercept and *p*-value mentioned (Supplementary Figure S11; Supplementary Figure S12) Horizontal pleiotropy was indicated in the MR analysis of LUAD and LUSQ, according to the result of the global test in MR PRESSO. (Table 5). Rs76145129 was identified as the outlying SNP in LUSQ. However, the removal of this SNP brought no significant difference in the MR estimate.

## Discussion

In this study, we estimated the causality between insomnia and lung cancer. The genetic liability to insomnia was causally correlated with lung cancer incidence. The positive associations were also observed in LUAD and LUSQ.

Insomnia usually leads to circadian disruption which has been classified as probably carcinogenic to humans (Group 2A) by the IARC (Humans and International Agency for Research on, 2010). Moreover, circadian disruption can alter the secretion patterns of melatonin (Kim et al., 2015). Melatonin was reported to have multiple anti-tumor effect, by modulating cell cycle, stimulating cell differentiation, inducing apoptosis, inhibiting metastasis and angiogenesis, and activating immune system, which was also found among lung cancer patients (Mediavilla et al., 2010) (Du-Quiton et al., 2010; Bhattacharya et al., 2019; Gurunathan et al., 2021). However, we should note that the disruption of the circadian rhythm of melatonin secretion was observed mainly in chronic primary insomnia patients (Hajak et al., 1995). Additionally, lower sleep quality was associated with decreased level of Klotho, an aging-suppressing protein, which also inhibits lung cancer cell growth and promotes lung cancer cell apoptosis (Chen et al., 2010; Chen et al., 2012; Mochón-Benguigui et al., 2020).

Some intermediate phenotypes can mediate the association between insomnia and lung cancer. A bidirectional causal relationship has been found between insomnia and smoking (Gibson et al., 2019). Specifically, smoking initiation and cigarettes smoked per day were positively correlated with insomnia. And insomnia was also found to be a promoter of smoking heaviness and an obstructor of smoking cessation. Considering the carcinogenicity of smoking in lung cancer, tobacco consumption may be an important mediator between insomnia and lung cancer (Hecht, 2002). Insomnia patients whose sleep duration was short or long were at higher risk of obesity and central obesity (Cai et al., 2018). In another MR analysis by Gao et al., body mass index (BMI) was identified as a risk factor of lung cancer (Gao et al., 2016). The above two previous studies indicated the potential intermediate effect of obesity in the positive relationship between insomnia and lung cancer, as was found in this study. The potential mediation of tobacco consumption and high BMI still needs verification in further research. Yet, the currently uncertain mediation status should not detract the importance of management for insomnia as one way to decrease the lung cancer risk.

This is the first large-scale MR study to illustrate the causal relationship between the genetic liability to insomnia and lung cancer. First, this study demonstrated its great clinical significance. It is an important mission to identify and intervene modifiable risk factors of lung cancer, and finally reduce the incidence of lung cancer. With the utilization of MR analysis, the genetic susceptibility to insomnia was found to be a risk factor of lung cancer. We advocate medical intervention on insomnia, along with the advocacy of other healthy lifestyles, like cigarette cessation (Siegel et al., 2019). Second, several issues may confuse the result of observational study on the association between insomnia and lung cancer. In this MR analysis, we used genetic liability to insomnia as a proxy of exposure. The effect of confounders and inverse causality, which are common in observational study, were avoided in MR analysis (Fewell et al., 2007; Davey Smith and Hemani, 2014). With the utilization of SNPs as proxies of exposure, confounders can be avoided, as SNPs are randomly allocated in the population during gamete formation, serving as a mimic of the randomization in RCT. To our concern, the problem of inverse causality should be paid extra attention to in the study of the relationship between insomnia and lung cancer, because insomnia is a common mental disorder in lung cancer patients (Savard and Morin, 2001). Third, RCT has been regarded as a steady approach for the estimation of causality. However, in the assessment of the causality between insomnia and lung cancer, RCT is infeasible and unethical. MR analysis was used in this study instead. Last but not least, no violation of the assumptions of MR analysis was observed in this study. The sample size was also large enough to support our findings.

However, there are still some limitations in this study. First, the two GWASs utilized were based on the United Kingdom population. The application of our conclusion in other populations may cause some unknown biases. Second, with the application of GWAS summary data, we couldn’t make stratification of the sample, because we didn’t have access to the individual characteristics of the studied population, like age, smoking status and so on. Third, the genetic liability to insomnia was used in this study as a proxy of insomnia, but it did not mean that every individual with those SNPs would necessarily suffer from insomnia. While insomnia is a disorder closely correlated with physical illness, behavioral factors, environment and medications (Kamel and Gammack, 2006). Researchers should be cautious when interpreting our result. Fourth, bidirectional MR analysis between insomnia and lung cancer and multivariable MR study were also infeasible, because of the limited data accessed. However, the result of this study was still rational because directional horizontal pleiotropy was not found in MR Egger and the distortion test did not deny our MR estimates even though the global test identified horizontal pleiotropy. MR analysis is an effective method in terms of causality estimation, a mimic of RCT (Smith and Ebrahim, 2003, 2004). Finally, the direct underlying mechanism is still unknown and needs further exploration or verification.

## Conclusion

In conclusion, the genetic susceptibility to insomnia was causally correlated with higher incidence of lung cancer, along with its histological subtype, LUAD and LUSQ. Effective intervention and management for insomnia were recommended to improve the sleep quality itself and to prevent lung cancer. Moreover, regular screening for lung cancer may be beneficial for patients with insomnia. However, further prospective studies are warranted to confirm the results and clarify the underlying mechanism.

## Data Availability

The datasets presented in this study can be found in online repositories. The names of the repository/repositories and accession number(s) can be found in the article/Supplementary Material.

## Abbreviations

BMI, body mass index; CI, confidence interval; GWAS, Genome-wide Association Study; IARC, International Agency for Research on Cancer; ILCCO, International Lung Cancer Consortium; IVW, inverse variance weighted; LUAD, lung adenocarcinoma; LUSQ, lung squamous cell carcinoma; MR, Mendelian randomization; OR, odds ratio; RCT, randomized controlled trial; SNPs, single-nucleotide polymorphisms; UKB, United Kingdom Biobank.

## References

[B1] Bhattacharya S. Patel K. K. Dehari D. Agrawal A. K. Singh S. (2019). Melatonin and its Ubiquitous Anticancer Effects. Mol. Cel Biochem 462 (1-2), 133–155. 10.1007/s11010-019-03617-5 31451998

[B2] Boef A. G. C. Dekkers O. M. le Cessie S. (2015). Mendelian Randomization Studies: a Review of the Approaches Used and the Quality of Reporting. Int. J. Epidemiol. 44 (2), 496–511. 10.1093/ije/dyv071 25953784

[B3] Bowden J. Davey Smith G. Burgess S. (2015). Mendelian Randomization with Invalid Instruments: Effect Estimation and Bias Detection through Egger Regression. Int. J. Epidemiol. 44 (2), 512–525. 10.1093/ije/dyv080 26050253 PMC4469799

[B4] Brion M.-J. A. Shakhbazov K. Visscher P. M. (2013). Calculating Statistical Power in Mendelian Randomization Studies. Int. J. Epidemiol. 42 (5), 1497–1501. 10.1093/ije/dyt179 24159078 PMC3807619

[B5] Burgess S. Scott R. A. Scott R. A. Timpson N. J. Davey Smith G. Thompson S. G. (2015). Using Published Data in Mendelian Randomization: a Blueprint for Efficient Identification of Causal Risk Factors. Eur. J. Epidemiol. 30 (7), 543–552. 10.1007/s10654-015-0011-z 25773750 PMC4516908

[B6] Burgess S. Thompson S. G. (2017). Interpreting Findings from Mendelian Randomization Using the MR-Egger Method. Eur. J. Epidemiol. 32 (5), 377–389. 10.1007/s10654-017-0255-x 28527048 PMC5506233

[B7] Cai G.-H. Theorell-Haglöw J. Janson C. Svartengren M. Elmståhl S. Lind L. (2018). Insomnia Symptoms and Sleep Duration and Their Combined Effects in Relation to Associations with Obesity and central Obesity. Sleep Med. 46, 81–87. 10.1016/j.sleep.2018.03.009 29773216

[B8] Chen B. Ma X. Liu S. Zhao W. Wu J. (2012). Inhibition of Lung Cancer Cells Growth, Motility and Induction of Apoptosis by Klotho, a Novel Secreted Wnt Antagonist, in a Dose-dependent Manner. Cancer Biol. Ther. 13 (12), 1221–1228. 10.4161/cbt.21420 22922788 PMC3469480

[B9] Chen B. Wang X. Zhao W. Wu J. (2010). Klotho Inhibits Growth and Promotes Apoptosis in Human Lung Cancer Cell Line A549. J. Exp. Clin. Cancer Res. 29, 99. 10.1186/1756-9966-29-99 20642846 PMC2912837

[B10] Chen P. Wang C. Song Q. Chen T. Jiang J. Zhang X. (2019). Impacts of Sleep Duration and Snoring on the Risk of Esophageal Squamous Cell Carcinoma. J. Cancer 10 (9), 1968–1974. 10.7150/jca.30172 31205556 PMC6548174

[B11] Davey Smith G. Ebrahim S. (2003). 'Mendelian Randomization': Can Genetic Epidemiology Contribute to Understanding Environmental Determinants of Disease? Int. J. Epidemiol. 32 (1), 1–22. 10.1093/ije/dyg070 12689998

[B12] Davey Smith G. Hemani G. (2014). Mendelian Randomization: Genetic Anchors for Causal Inference in Epidemiological Studies. Hum. Mol. Genet. 23 (R1), R89–R98. 10.1093/hmg/ddu328 25064373 PMC4170722

[B13] Du-Quiton J. Wood P. A. Burch J. B. Grutsch J. F. Gupta D. Tyer K. (2010). Actigraphic Assessment of Daily Sleep-Activity Pattern Abnormalities Reflects Self-Assessed Depression and Anxiety in Outpatients with Advanced Non-small Cell Lung Cancer. Psycho-oncology 19 (2), 180–189. 10.1002/pon.1539 19199317

[B14] Fewell Z. Davey Smith G. Sterne J. A. C. (2007). The Impact of Residual and Unmeasured Confounding in Epidemiologic Studies: a Simulation Study. Am. J. Epidemiol. 166 (6), 646–655. 10.1093/aje/kwm165 17615092

[B15] Gao C. Patel C. J. Michailidou K. Peters U. Gong J. Schildkraut J. (2016). Mendelian Randomization Study of Adiposity-Related Traits and Risk of Breast, Ovarian, Prostate, Lung and Colorectal Cancer. Int. J. Epidemiol. 45 (3), 896–908. 10.1093/ije/dyw129 27427428 PMC6372135

[B16] Gibson M. Munafò M. R. Taylor A. E. Treur J. L. (2019). Evidence for Genetic Correlations and Bidirectional, Causal Effects between Smoking and Sleep Behaviors. Nicotine Tob. Res. 21 (6), 731–738. 10.1093/ntr/nty230 30365022 PMC6528151

[B17] Gurunathan S. Qasim M. Kang M.-H. Kim J.-H. (2021). Role and Therapeutic Potential of Melatonin in Various Type of Cancers. Onco Targets Ther. 14, 2019–2052. 10.2147/OTT.S298512 33776451 PMC7987311

[B18] Hajak G. Rodenbeck A. Staedt J. Bandelow B. Huether G. Rüther E. (1995). Nocturnal Plasma Melatonin Levels in Patients Suffering from Chronic Primary Insomnia. J. Pineal Res. 19 (3), 116–122. 10.1111/j.1600-079x.1995.tb00179.x 8750344

[B19] Hecht S. S. (2002). Cigarette Smoking and Lung Cancer: Chemical Mechanisms and Approaches to Prevention. Lancet Oncol. 3 (8), 461–469. 10.1016/s1470-2045(02)00815-x 12147432

[B20] Hemani G. Zheng J. Elsworth B. Wade K. H. Haberland V. Baird D. (2018). The MR-Base Platform Supports Systematic Causal Inference across the Human Phenome. Elife 7, e34408. 10.7554/eLife.34408 29846171 PMC5976434

[B21] Herbst R. S. Morgensztern D. Boshoff C. (2018). The Biology and Management of Non-small Cell Lung Cancer. Nature 553 (7689), 446–454. 10.1038/nature25183 29364287

[B22] Humans I. W. G. o. t. E. o. C. R. t. International Agency for Research on C. (2010). Painting, Firefighting, and Shiftwork. Geneva, Switzerland: World Health Organization.

[B23] Jansen P. R. Watanabe K. Watanabe K. Stringer S. Skene N. Bryois J. (2019). Genome-wide Analysis of Insomnia in 1,331,010 Individuals Identifies New Risk Loci and Functional Pathways. Nat. Genet. 51 (3), 394–403. 10.1038/s41588-018-0333-3 30804565

[B24] Kakizaki M. Kuriyama S. Sone T. Ohmori-Matsuda K. Hozawa A. Nakaya N. (2008). Sleep Duration and the Risk of Breast Cancer: the Ohsaki Cohort Study. Br. J. Cancer 99 (9), 1502–1505. 10.1038/sj.bjc.6604684 18813313 PMC2579702

[B25] Kamel N. S. Gammack J. K. (2006). Insomnia in the Elderly: Cause, Approach, and Treatment. Am. J. Med. 119 (6), 463–469. 10.1016/j.amjmed.2005.10.051 16750956

[B26] Kim T. W. Jeong J.-H. Hong S.-C. (2015). The Impact of Sleep and Circadian Disturbance on Hormones and Metabolism. Int. J. Endocrinol. 2015, 1–9. 10.1155/2015/591729 PMC4377487 25861266

[B27] Lawlor D. A. (2016). Commentary: Two-Sample Mendelian Randomization: Opportunities and Challenges. Int. J. Epidemiol. 45 (3), 908–915. 10.1093/ije/dyw127 27427429 PMC5005949

[B28] Luojus M. K. Lehto S. M. Tolmunen T. Erkkilä A. T. Kauhanen J. (2014). Sleep Duration and Incidence of Lung Cancer in Ageing Men. BMC Public Health 14, 295. 10.1186/1471-2458-14-295 24684747 PMC4229981

[B29] Mediavilla M. D. Sanchez-Barcelo E. J. Tan D. X. Manchester L. Reiter R. J. (2010). Basic Mechanisms Involved in the Anti-cancer Effects of Melatonin. Curr. Med. Chem. 17 (36), 4462–4481. 10.2174/092986710794183015 21062257

[B30] Mochón-Benguigui S. Carneiro-Barrera A. Castillo M. J. Amaro-Gahete F. J. (2020). Is Sleep Associated with the S-Klotho Anti-aging Protein in Sedentary Middle-Aged Adults? the FIT-AGEING Study. Antioxidants 9 (8), 738. 10.3390/antiox9080738 PMC7463654 32806634

[B31] Morin C. M. Drake C. L. Harvey A. G. Krystal A. D. Manber R. Riemann D. (2015). Insomnia Disorder. Nat. Rev. Dis. Primers 1, 15026. 10.1038/nrdp.2015.26 27189779

[B32] Roth T. (2007). Insomnia: Definition, Prevalence, Etiology, and Consequences. J. Clin. Sleep Med. 3 (5 Suppl. l), S7–S10. 10.5664/jcsm.26929 17824495 PMC1978319

[B33] Savard J. Morin C. M. (2001). Insomnia in the Context of Cancer: A Review of a Neglected Problem. J. Clin. Oncol. 19 (3), 895–908. 10.1200/jco.2001.19.3.895 11157043

[B34] Siegel R. L. Miller K. D. Jemal A. (2019). Cancer Statistics, 2019. CA A. Cancer J. Clin. 69 (1), 7–34. 10.3322/caac.21551 30620402

[B35] Smith G. D. Ebrahim S. (2004). Mendelian Randomization: Prospects, Potentials, and Limitations. Int. J. Epidemiol. 33 (1), 30–42. 10.1093/ije/dyh132 15075143

[B36] Smith G. D. Timpson N. Ebrahim S. (2008). Strengthening Causal Inference in Cardiovascular Epidemiology through Mendelian Randomization. Ann. Med. 40 (7), 524–541. 10.1080/07853890802010709 18608114

[B37] Verbanck M. Chen C.-Y. Neale B. Do R. (2018). Detection of Widespread Horizontal Pleiotropy in Causal Relationships Inferred from Mendelian Randomization between Complex Traits and Diseases. Nat. Genet. 50 (5), 693–698. 10.1038/s41588-018-0099-7 29686387 PMC6083837

[B38] Wang Y. McKay J. D. Rafnar T. Wang Z. Timofeeva M. N. Broderick P. (2014). Rare Variants of Large Effect in BRCA2 and CHEK2 Affect Risk of Lung Cancer. Nat. Genet. 46 (7), 736–741. 10.1038/ng.3002 24880342 PMC4074058

