# Supplementary material for: Genetic Liability to Insomnia and Lung Cancer Risk: A Mendelian Randomization Analysis
Source: Front Genet. 2021 Dec 1;12:756908. doi: 10.3389/fgene.2021.756908 (PMC8672094; doi:10.3389/fgene.2021.756908)
Supplement: Supplementary file 1 [file DataSheet1.PDF]

Genetic Liability to Insomnia and Lung Cancer Risk: a Mendelian Randomization Analysis

Contents:

Supplementary figure 1. Mendelian randomization regression slopes of the MR analysis between insomnia and lung cancer;

Supplementary figure 2. Single SNP analysis in the MR analysis between insomnia and lung cancer;

Supplementary figure 3. Leave-one-out analysis in the MR analysis between insomnia and lung cancer;

Supplementary figure 4. Funnel plot in the MR Egger analysis between insomnia and lung cancer;

Supplementary figure 5. Mendelian randomization regression slopes of the MR analysis between insomnia and lung adenocarcinoma;

Supplementary figure 6. Mendelian randomization regression slopes of the MR analysis between insomnia and lung squamous cell carcinoma;

Supplementary figure 7. Single SNP analysis in the MR analysis between insomnia and lung adenocarcinoma;

Supplementary figure 8. Single SNP analysis in the MR analysis between insomnia and lung squamous cell carcinoma;

Supplementary figure 9. Leave-one-out analysis in the MR analysis between insomnia and lung adenocarcinoma;

Supplementary figure 10. Leave-one-out analysis in the MR analysis between insomnia and lung squamous cell carcinoma;

Supplementary figure 11. Funnel plot in the MR Egger analysis between insomnia and lung adenocarcinoma;

Supplementary figure 12. Funnel plot in the MR Egger analysis between insomnia and lung squamous cell carcinoma.

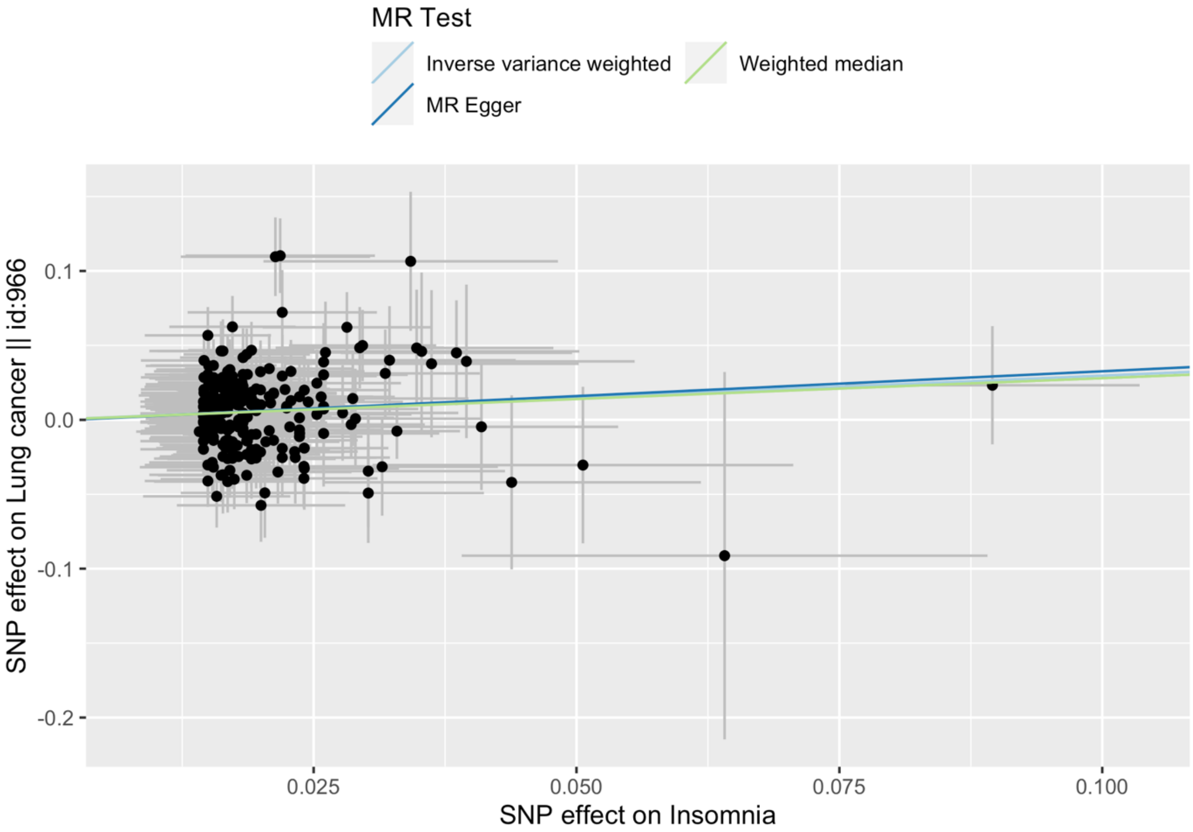

Supplementary figure 1. Mendelian randomization regression slopes of the MR analysis between insomnia and lung cancer.

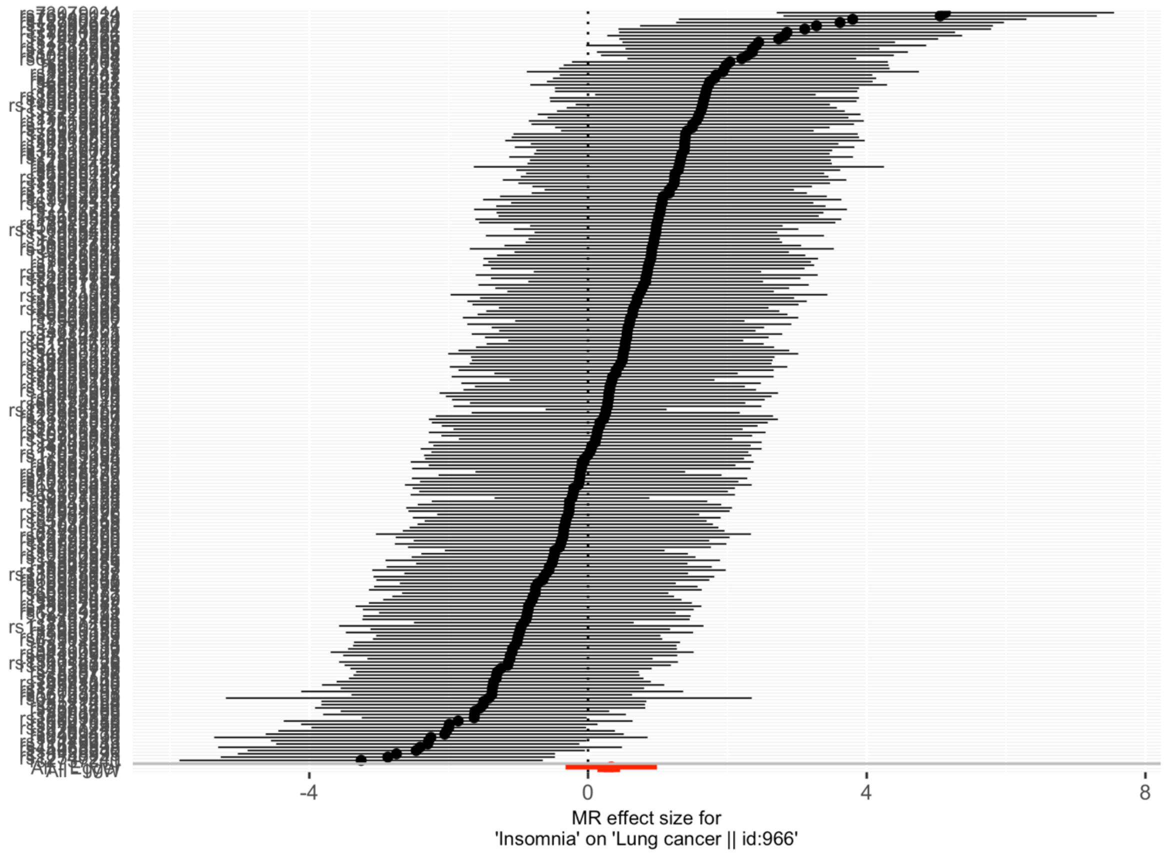

Supplementary figure 2. Single SNP analysis in the MR analysis between insomnia and lung cancer.

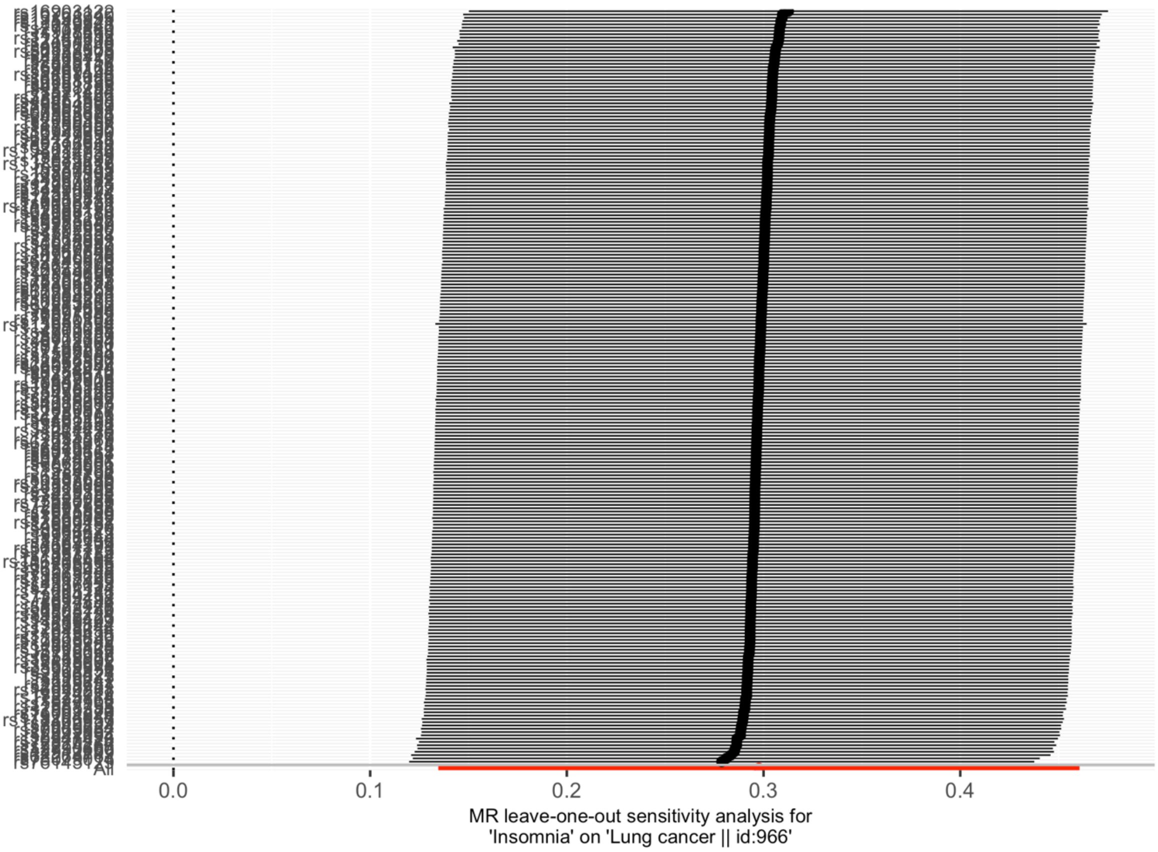

Supplementary figure 3. Leave-one-out analysis in the MR analysis between insomnia and lung cancer.

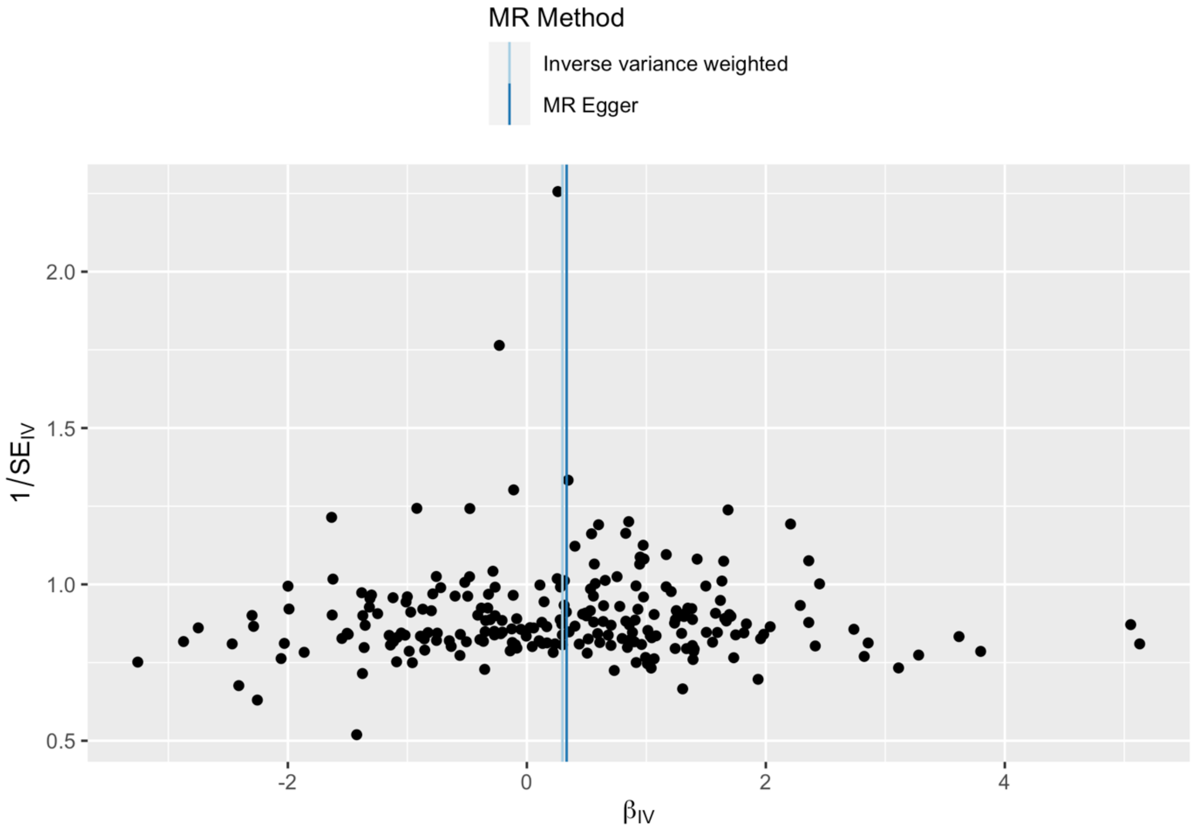

Supplementary figure 4. Funnel plot in the MR Egger analysis between insomnia and lung cancer.

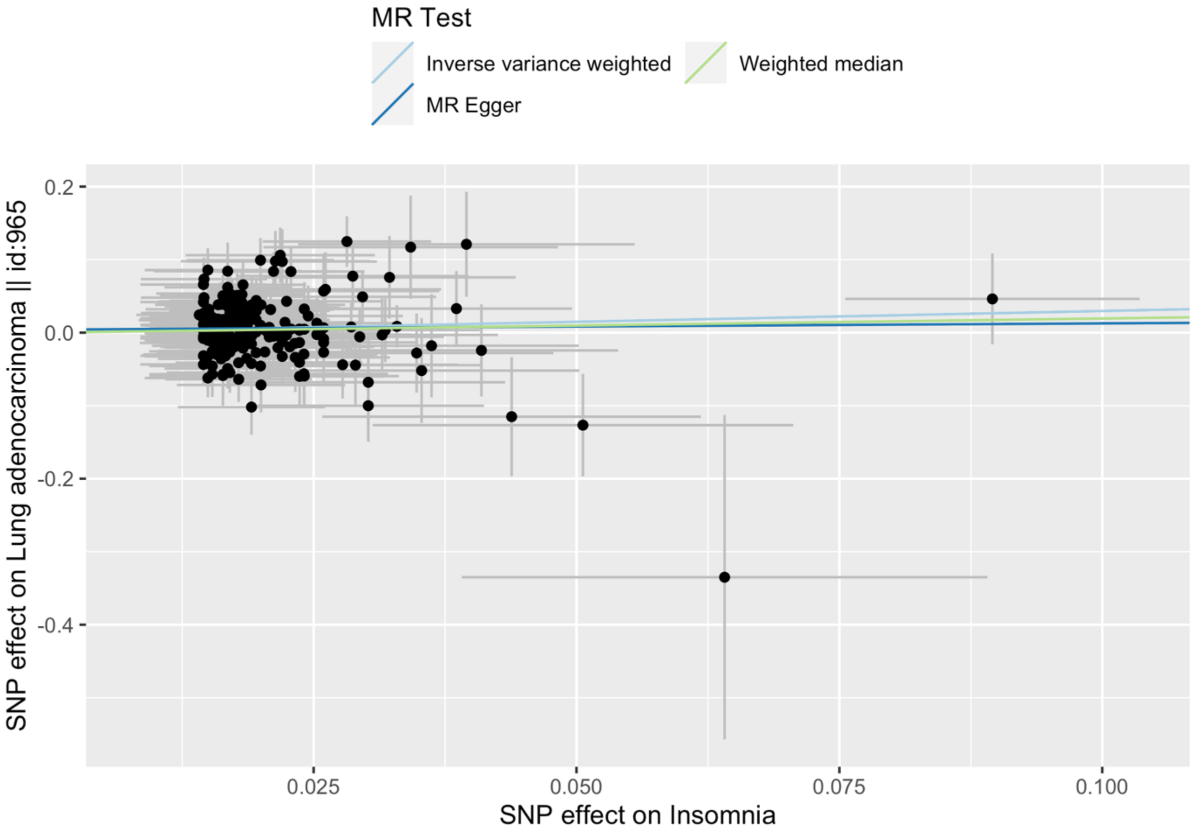

Supplementary figure 5. Mendelian randomization regression slopes of the MR analysis between insomnia and lung adenocarcinoma.

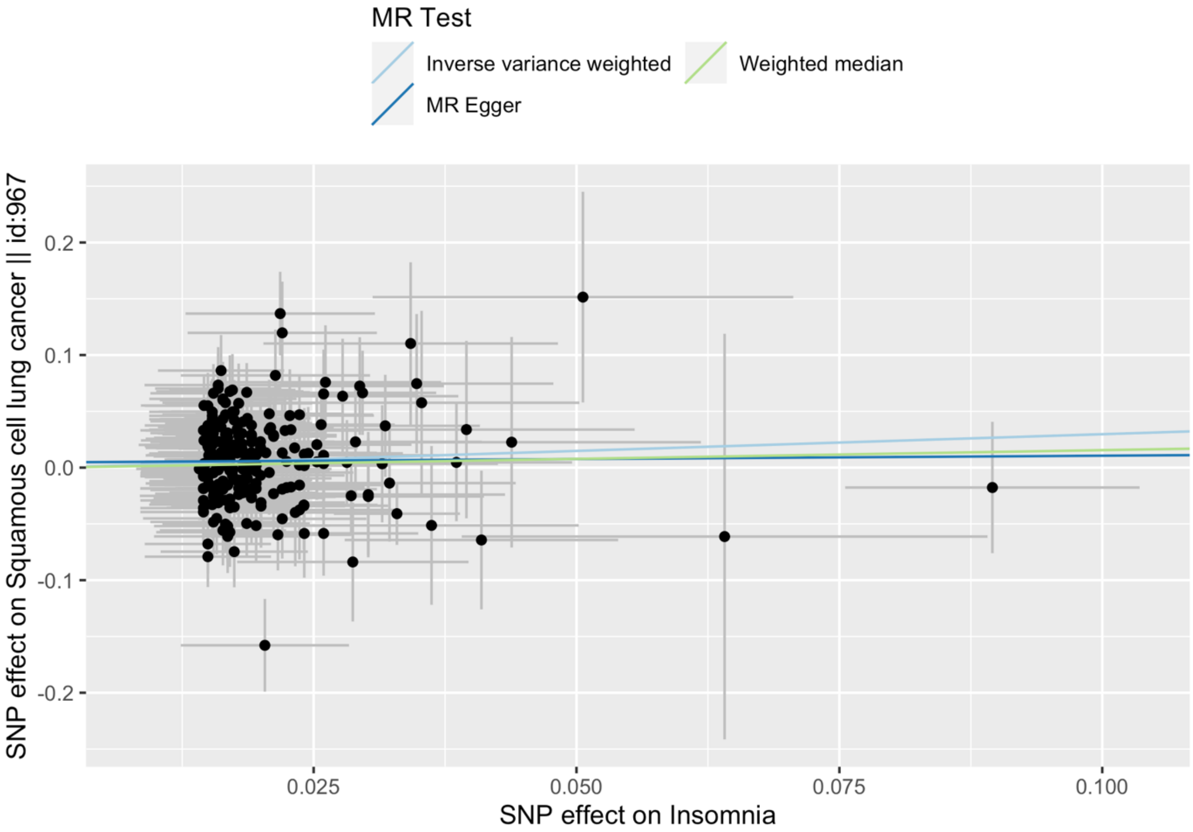

Supplementary figure 6. Mendelian randomization regression slopes of the MR analysis between insomnia and lung squamous cell carcinoma.

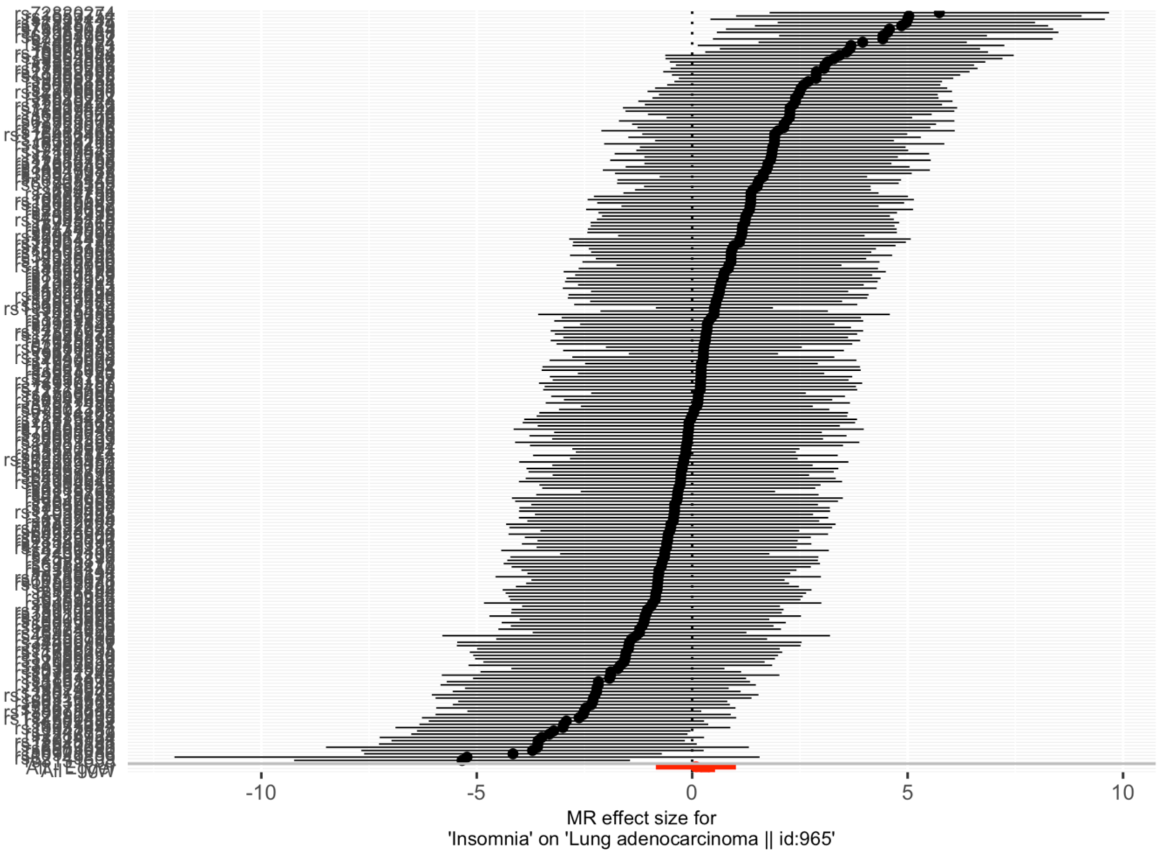

Supplementary figure 7. Single SNP analysis in the MR analysis between insomnia and lung adenocarcinoma.

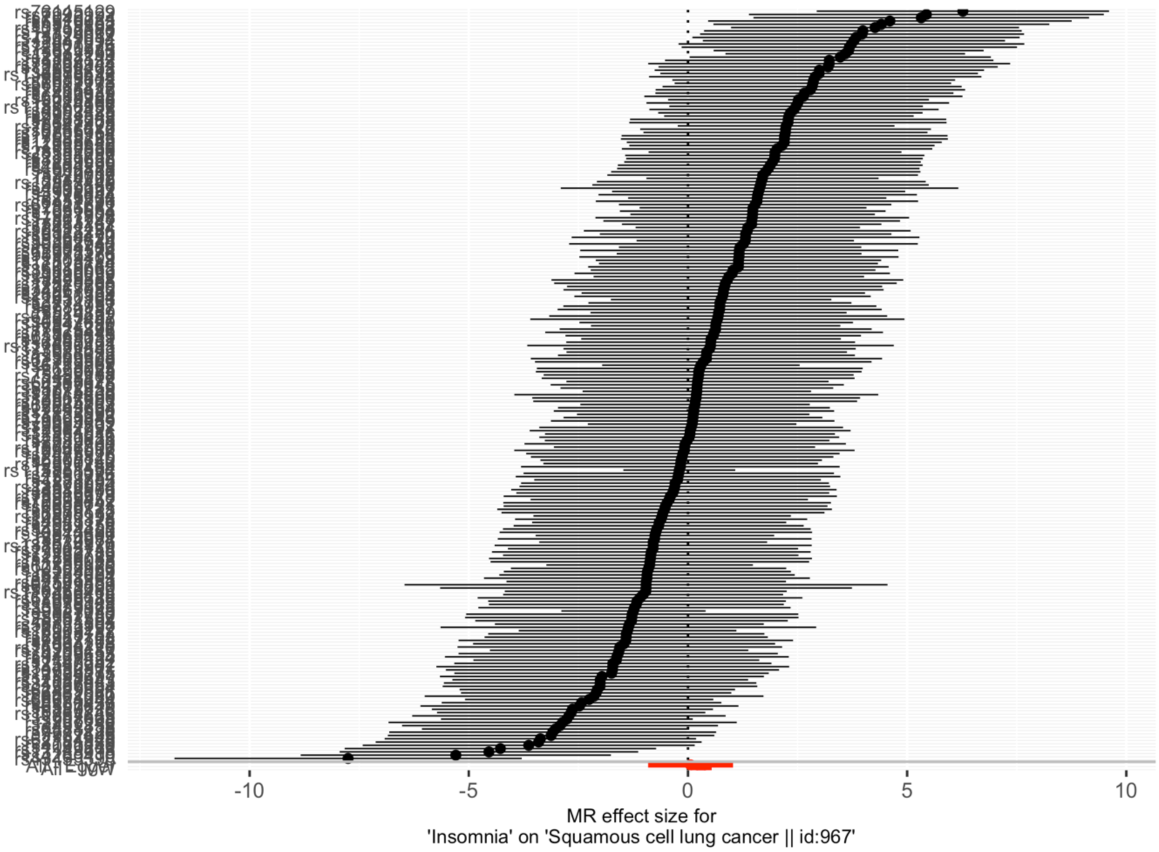

Supplementary figure 8. Single SNP analysis in the MR analysis between insomnia and lung squamous cell carcinoma.

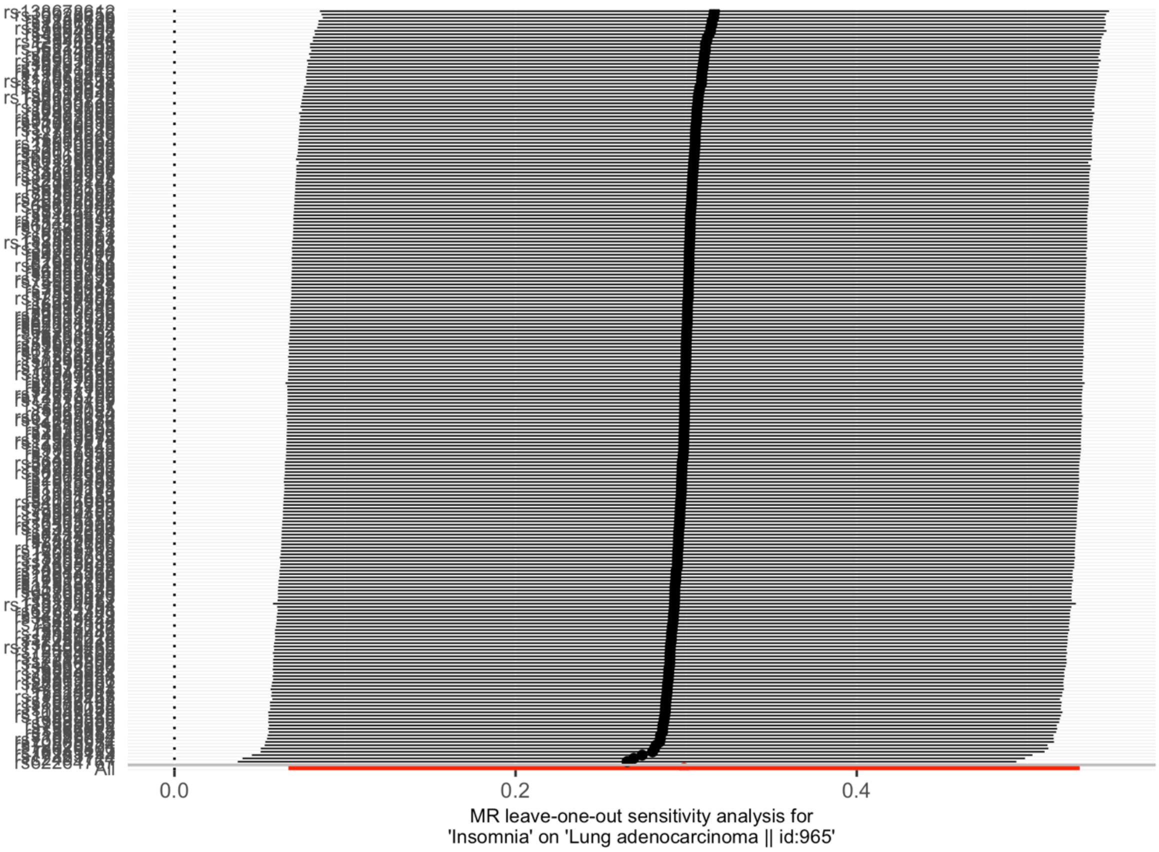

Supplementary figure 9. Leave-one-out analysis in the MR analysis between insomnia and lung adenocarcinoma.

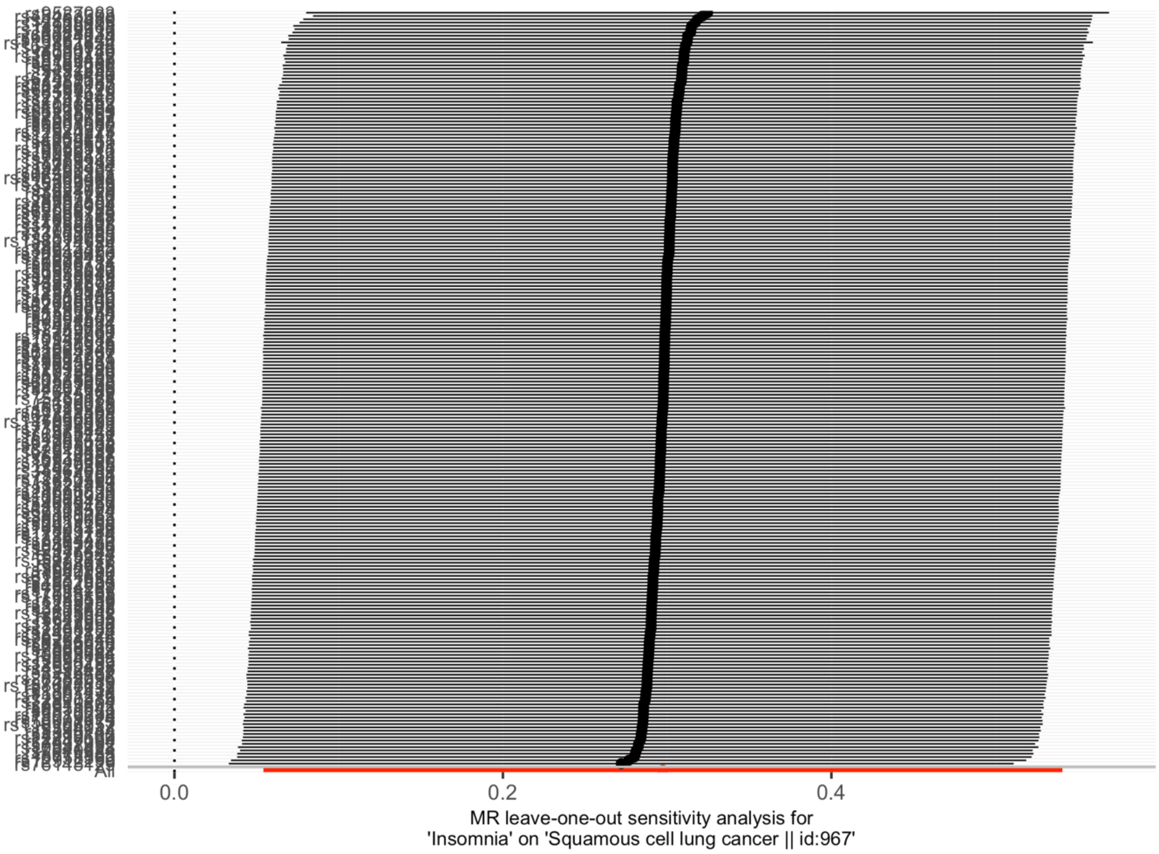

Supplementary figure 10. Leave-one-out analysis in the MR analysis between insomnia and lung squamous cell carcinoma.

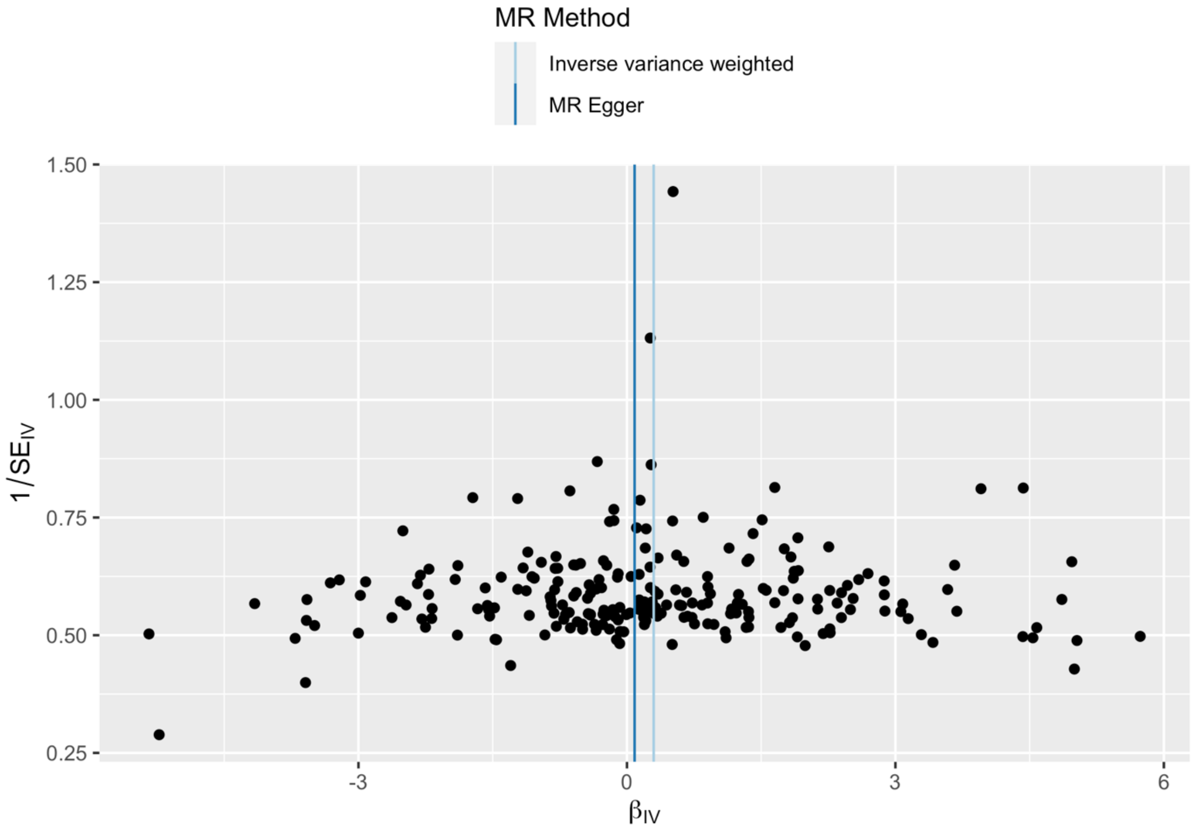

Supplementary figure 11. Funnel plot in the MR Egger analysis between insomnia and lung adenocarcinoma.

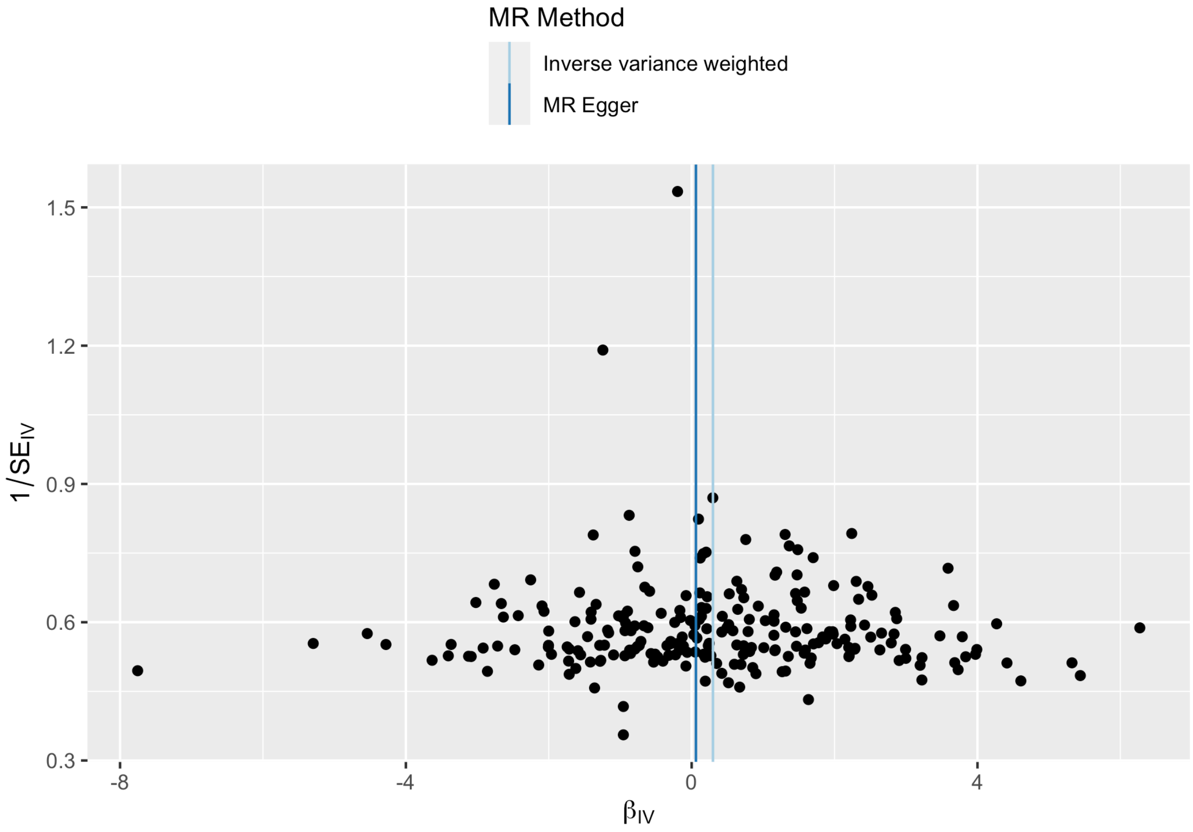

Supplementary figure 12. Funnel plot in the MR Egger analysis between insomnia and lung squamous cell carcinoma.
